# Oxygen saturation-dependent effects on blood transverse relaxation at low fields

**DOI:** 10.1007/s10334-021-00993-2

**Published:** 2022-02-02

**Authors:** Dion G. Thomas, Petrik Galvosas, Yu-Chieh Tzeng, Freya G. Harrison, Mary J. Berry, Paul D. Teal, Graham A. Wright, Sergei Obruchkov

**Affiliations:** 1 School of Chemical and Physical Sciences and MacDiarmid Institute for Advanced Materials, Victoria University of Wellington, Wellington, New Zealand; 2 Centre for Translational Research, University of Otago, Wellington, New Zealand; 3 Centre for Translational Research and Department of Paediatrics and Child Health, University of Otago, Wellington, New Zealand; 4 School of Engineering and Computer Science, Victoria University of Wellington, Wellington, New Zealand; 5 Sunnybrook Research Institute and University of Toronto, Toronto, ON Canada; 6 Robinson Research Institute, Victoria University of Wellington, Wellington, New Zealand

**Keywords:** Blood $\textit{T}_{2}$, Oxygenation, Relaxometry, CPMG echo interval, Low field

## Abstract

**Objective:**

Blood oxygenation can be measured using magnetic resonance using the paramagnetic effect of deoxy-haemoglobin, which decreases the $\textit{T}_{2}$ relaxation time of blood. This $\textit{T}_{2}$ contrast has been well characterised at the $\textit{B}_{{0}}$ fields used in MRI (1.5 T and above). However, few studies have characterised this effect at lower magnetic fields. Here, the feasibility of blood oximetry at low field based on $\textit{T}_{2}$ changes that are within a physiological relevant range is explored. This study could be used for specifying requirements for construction of a monitoring device based on low field permanent magnet systems.

**Methods:**

A continuous flow circuit was used to control parameters such as oxygen saturation and temperature in a sample of blood. It flowed through a variable field magnet, where CPMG experiments were performed to measure its $\textit{T}_{2}$. In addition, the oxygen saturation was monitored by an optical sensor for comparison with the $\textit{T}_{2}$ changes.

**Results:**

These results show that at low $\textit{B}_{{0}}$ fields, the change in blood $\textit{T}_{2}$ due to oxygenation is small, but still detectable. The data measured at low fields are also in agreement with theoretical models for the oxy-deoxy $\textit{T}_{2}$ effect.

**Conclusion:**

$\textit{T}_{2}$ changes in blood due to oxygenation were observed at fields as low as 0.1 T. These results suggest that low field NMR relaxometry devices around 0.3 T could be designed to detect changes in blood oxygenation.

## Introduction

The $\textit{T}_{2}$ of blood is sensitive to the oxygen saturation, producing a useful MRI contrast. This has been applied at the $\textit{B}_{{0}}$ fields used in typical MRI systems, but the complexity and cost of these systems limit their accessibility. Moving to lower field presents new opportunities for applying MRI techniques, and helps to make magnetic resonance technology more accessible [1, 2]. Magnetic resonance systems that operate at lower fields than conventional scanners have been developed to achieve this goal [3, 4]. MRI contrast mechanisms have also been applied in portable low field MR relaxometry devices, which may unlock new uses of magnetic resonance for point of care diagnosis and monitoring [5–10].

Blood oxygen saturation (${\textit{s}}\text{O}_{2}$) affects the transverse relaxation time ($\textit{T}_{2}$) of whole blood due to the effect of deoxy-haemoglobin concentrated inside the red blood cells [11]. Deoxy-haemoglobin is more paramagnetic than oxy-haemoglobin, meaning that the susceptibility change between the intra- and extra-cellular space becomes greater as the oxygen saturation decreases. Susceptibility differences create an inhomogeneous field, which dephases protons in the water surrounding the cells. As oxygen saturation falls, the $\textit{T}_{2}$ appears to decrease as the susceptibility change becomes stronger. By calibrating $\textit{T}_{2}$ and oxygen saturation, this effect has been used in vivo to measure oxygen saturation and oxygen extraction fraction [12–14].

Working at lower $\textit{B}_{{0}}$ field has been shown to change the behaviour of $\textit{T}_{{1}}$ and $\textit{T}_{2}$ contrasts [15]. In this study, we investigate how changes in $\textit{B}_{{0}}$ affect the relationship between the oxygen saturation of blood and its $\textit{T}_{2}$. This $\textit{T}_{2}$ change is known to be dependent on $\textit{B}_{{0}}$ field strength as it is caused by changes in magnetic susceptibility, which causes greater effects at higher fields. While the effect has been well characterised at fields over 1 T [16–23], there are few studies at fields below this. Importantly, previous low field studies have been limited to non-physiological extreme levels of oxygenation, measuring only fully oxygenated (100%) and deoxygenated (0%) blood samples [24–26]. These levels are well outside the ranges typically seen in humans under a range of pathological conditions (e.g., hypovolaemic shock, sepsis, ischaemic stroke). The extent to which clinically relevant $\textit{T}_{2}$ changes can reliably differentiate between moderate (80–90%) and severe $(<80\%)$ levels of de-saturation under low field conditions has been unclear. The application of $\textit{T}_{2}$ contrast for monitoring application would require this sensitivity to be known across all ranges of blood oxygenation.

In this study, we explore whether $\textit{T}_{2}$ changes due to dynamic changes in blood oxygenation can be detected using low field or single-sided MR relaxometry for use in physiological monitoring. We measured the dependence of blood $\textit{T}_{2}$ on oxygen saturation at five field strengths from 0.1 to 1.0 T (0.12, 0.24, 0.3, 0.5, and 1 T) using a continuous flow circuit to generate smooth ramps in the oxygen saturation profile [27]. These fields were chosen to represent the range of $\textit{B}_{{0}}$ fields available using permanent magnet single-sided MR systems, as well as newly developed MRI systems that operate at low fields. This setup allowed us to evaluate the sensitivity of this $\textit{T}_{2}$ contrast to dynamic changes in oxygenation in a physiologically realistic range, without the confounding influences of erythrocyte sedimentation, in a similar way to the setup described by Meyer [16]. $\textit{T}_{2}$ was measured using the Carr–Purcell–Meiboom–Gill (CPMG) experiment with five different inter-echo intervals: 1ms, 5ms, 8ms, 10ms and 20ms. In addition, we investigated whether existing models for the oxygenation effect describe the change in $\textit{T}_{2}$ at low field by measuring $\textit{T}_{2}$ with a range of inter-echo intervals. In particular, the Exchange model using the Luz–Meiboom equation, and the Diffusion model proposed by Jensen and Chandra are tested. These models have been used in the literature to help improve the accuracy of MRI-based oximetry using this $\textit{T}_{2}$ contrast [28].

## Methods

*Blood* Four samples of whole blood (approximately 450 ml) were collected from healthy volunteers by venipuncture of the antecubital vein. The collection of blood for this study was approved by the New Zealand Central Health and Disability Ethics Committee. One sample of blood was collected from each participant. Blood was collected into CPD anticoagulant bags and equipment (Leukotrap WB, Haemonetics, Braintree MA USA) and filtered through a leukocyte reduction filter before storage at 4 ${}^{\circ }\text{C}$. Blood used in experiments was no more than 14 days old before experiments, with no further processing. The collection process meant that the haematocrit was typically 0.34–0.38 rather than the typical value of 0.4, as it was diluted by the anticoagulant/ preservative solution. Each sample of blood was used for a single experiment, with one for each $\textit{B}_{{0}}$ field strength, with the exception of the 1.0 T and 0.5 T experiments, where the sample was retained in the circuit and re-used.

**Fig. 1:**
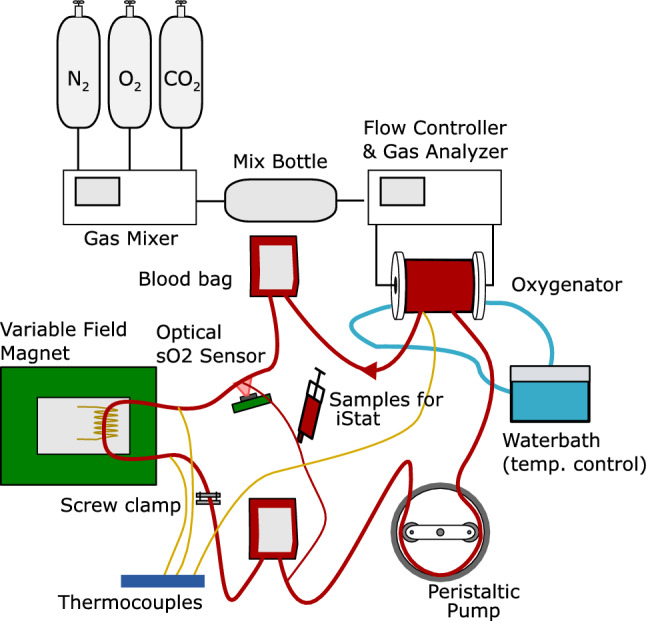
Schematic of flow circuit used for blood experiments

*Circuit* To measure at a range of oxygenation levels, a continuous flow circuit was used to slowly ramp between oxygenated and deoxygenated states. This system is similar to the circuits used for cardiopulmonary bypass, including a Stockert SIII roller pump (Sorin Group, Munich Germany) and an Affinity Pixie oxygenator unit (Medtronic, Minneapolis MN USA) and is shown in Fig. 1. The circuit itself is constructed from $\frac{1}{4}~''$ medical grade PVC tube. The tubing and oxygenator had a volume of 60 ml.

The oxygenation level was controlled by altering the composition of gases flowing through the oxygenator using a precision gas mixer (MapMix Provectus, Dansensor, Ringsted Denmark). To obtain fully oxygenated blood, the gas mix was set to 21% $\text{O}_{2}$, 5% $\text{CO}_{2}$ and 74% $\text{N}_{2}$, while for deoxygenated blood, the mix was set to 0% $\text{O}_{2}$, 5% $\text{CO}_{2}$ and 95% $\text{N}_{2}$, as described in [16]. An intermediate level of oxygenation (approximately 50%) was reached with 5% $\text{O}_{2}$, and was used to slow the rate of oxygenation, which could happen very rapidly.

The small fraction of $\text{CO}_{2}$ was also added to maintain physiological pH levels. The gas mix was measured by a gas analyser unit (MapChek 3, Dansensor Ringsted Denmark) before flowing into the oxygenator.

Temperature was controlled by flowing 37 ${}^{\circ }\text{C}$ water from a waterbath through the oxygenator to warm the blood as it flowed through the circuit. Blood temperature was measured by a pair of thermocouples in the circuit at the entrance and exit of the magnet. This setup produced a blood temperature between 27 and 30 ${}^{\circ }\text{C}$ at the entrance of the magnet, dropping by 2 ${}^{\circ }\text{C}$ on average after flowing through the probe. The upper section of the circuit was insulated with foam and the bore of the magnet was also warmed to limit the temperature drop before the blood reached the probe.

To produce a steady flow through the circuit, the blood flowed between the two bags under gravity, with a flow rate of 1–2 cm/s controlled by the screw clamp. Blood was then pumped from the lower bag, through the oxygenator and into the upper bag by the roller pump. The pump speed was set to maintain a constant level in the upper bag. With steady flow in the circuit, this did not affect the $\textit{T}_{2}$ we measured, although fluctuations in the flow rate occurred when the bags or pump was adjusted.

*Optical sensor* The oxygen saturation was measured using an MAX30102 two wavelength pulse oximetry module (Maxim Integrated, San Diego CA USA) which allowed the raw red (660 nm) and infrared (880 nm) signal intensities to be continuously output to an Arduino and recorded. This was clipped onto a polystyrene tube joint and positioned to measure the blood just before it flowed into the magnet. To convert the signal intensities into an oxygen saturation, the sensor was calibrated against an iStat blood gas analyzer (Abbott, CG8+ cartridges) by withdrawing 1 ml samples of blood as the oxygen saturation was ramped down and correlating the samples’ ${\textit{s}}\text{O}_{2}$ with the optical data. As suggested by Wieben [29], a quadratic calibration equation for the ratio of the two light intensities (*R* = red/IR) was found to be a good fit to these samples. This calibration curve was within 5% of the ${\textit{s}}\text{O}_{2}$ measured on the iStat.

*NMR* NMR experiments were run using a variable field cryogenic-free magnet (Cryogenic, UK), set to fields of 0.1–1.0 T. The magnet also includes a 3 axis gradient coil set, used for shimming and measuring the flow rate by pulsed gradient spin echo (PGSE). Experiments were run using a Kea 2 console (Magritek, Wellington NZ) and an external radio frequency (RF) amplifier. Three 2-cm-long solenoid coils were custom built to work at the range of frequencies used in these experiments.

$\textit{T}_{2}$ was measured using a CPMG experiment with a four scan phase cycle with a $T_R$ of 1.2 s. The RF power was adjusted for the 90${}^{\circ }$ and 180${}^{\circ }$ pulses to give a pulse length of 20 $\upmu \text{s}$. The CPMG experiments produce an echo train which is acquired and fit to a monoexponential decay to obtain a $\textit{T}_{2}$ for the sample. In the main part of the experiment, five inter-echo intervals (1 ms, 5 ms, 8 ms, 10 ms and 20 ms) were measured sequentially, with the number of echoes adjusted to measure the signal decay out to 600 ms. Each set of 5 CPMG measurements, along with a PGSE velocity measurement took 45 s.

Once the blood reached a deoxygenated state, the main measurements were paused, a second series of CPMG experiments were run to test the dependence of $\textit{T}_{2}$ on the CPMG inter-echo interval. This experiment used 13 intervals between 0.5 and 20 ms, collecting 200 echoes for each interval.

*Experimental protocol* The four samples of blood were used for measurements at the five different field strengths: one sample for each of 0.12 T, 0.24 T and 0.3 T, and one sample for 0.5 T and 1.0 T. To warm the blood, and reach a fully oxygenated starting point, the bag was plugged into the circuit, and the pump switched on with the clamp fully open. At the start of the experiments, the screw clamp was tightened to produce a flow rate between 1 and 2 cm/s, measured by PGSE. This flow rate was maintained over the course of the experiment. $\textit{T}_{2}$ was measured to find a stable baseline, before the blood was deoxygenated by switching to the 0% $\text{O}_{2}$ gas mix. To reach a deoxygenated level required approximately 30–40 min of the blood flowing through the circuit and oxygenator. The gas mix was then set to 21% $\text{O}_{2}$, to reoxygenate the blood and observe the recovery in $\textit{T}_{2}$. During the data collection, we discovered that the oxygenation occurred extremely rapidly, so as the experimental protocol developed, we added a step to reach an intermediate level of oxygenation (approximately 50%) with 5% $\text{O}_{2}$, to slow the rate of oxygenation and increase the amount of data we could collect.

Measuring using a slow deoxygenation ramp was chosen to allow us to efficiently map across a wide range of ${\textit{s}}\text{O}_{2}$ values. It also means that the sensitivity to dynamic changes in ${\textit{s}}\text{O}_{2}$ can be observed, which is more representative of trend monitoring applications.

In addition, 3 ml samples were withdrawn from the circuit periodically to test the integrity of the blood. Blood plasma was separated by centrifugation, and spectra measured in a UV/Vis spectrometer (Shimadzu, Kyoto Japan). The Kahn method was used to convert these absorbance measurements into the concentration of free haemoglobin in the plasma fraction, which indicates the breakdown of red blood cells [30].

*Data analysis* Data were processed using Python in JuPyter notebooks. $\textit{T}_{2}$ values were found by fitting the time domain echo amplitudes to a monoexponential decay, as blood has a single relaxation time component. To remove the fast decaying signal of the PVC tube, the first 15 ms of each echo train was ignored in the fitting, while echoes after 360 ms were also ignored to reduce the effects of flow. The $\textit{T}_{2}$ values measured in each CPMG experiment was plotted versus measurement time, which allowed them to be correlated with data with the optical sensor. This produced a series of $\textit{T}_{2}$ values as a function of oxygenation for each inter-echo interval.

The rapid change in oxygen saturation as the blood was oxygenated meant that only data points measured during the deoxygenation section of the experiments were extracted for further analysis. The extracted $\textit{T}_{2}$ values were converted to a relaxation rate $\textit{R}_{{2}}$, then plotted as a function of ${\textit{s}}\text{O}_{2}$to produce calibration curves, similar to those of Lu et al. [14]. The relationship given in the literature is the quadratic dependence

1 $$\begin{aligned} \frac{1}{\textit{T}_{2}} (\textit{t}_{\mathrm{ec}}, \textit{B}_{{0}}, {\textit{s}}\text{O}_{2})&=\, \textit{R}_{{2}}(\textit{t}_{\mathrm{ec}}, \textit{B}_{{0}}, {\textit{s}}\text{O}_{2}) \\ &=\, \frac{1}{\textit{T}_{20}} + \mathrm {K}(\textit{t}_{\mathrm{ec}}, \textit{B}_{{0}}) (1-{\textit{s}}\text{O}_{2})^2 \end{aligned}$$

with constant *K* which depends on the field strength $\textit{B}_{{0}}$, and inter-echo interval $\textit{t}_{\mathrm{ec}}$, as well as the intrinsic $\textit{T}_{2}$ of blood $\textit{T}_{20}$. These calibration curves were used to directly convert the measured $\textit{T}_{2}$ maps into the blood saturation ${\textit{s}}\text{O}_{2}$.

*Model comparison* The CPMG inter-echo interval is another factor that affects the change in $\textit{T}_{2}$. As the time between echoes is increased, protons experience more decoherence during this period and the relaxation rate increases. Several models for the change in blood $\textit{T}_{2}$ have been published in the literature to explain this effect. Our experimental setup allowed us to explore this effect and test how well these models fit at low $\textit{B}_{{0}}$ fields. In particular, we looked at the exchange model (Luz–Meiboom equation) and the Jensen and Chandra diffusion model, which can be used to predict $\textit{T}_{2}$ based on the CPMG inter-echo interval [31].

In the exchange model, protons are assumed to exchange instantaneously between the intra- and extra-cellular compartments, which have slightly different $\textit{B}_{{0}}$ fields due to the haemoglobin [11, 24]. This exchange causes increased decoherence, and therefore a shorter $\textit{T}_{2}$. In this model, the $\textit{T}_{2}$ change due to deoxygenation is given by the Luz–Meiboom equation [18]:

2 $$\begin{aligned} \frac{1}{T_2} = \frac{1}{T_{20}} + \gamma ^2 K_0 \tau _{\mathrm{ex}} \left( 1 - \frac{2\tau _{\mathrm{ex}}}{t_{\mathrm{ec}}} \tanh {\frac{t_{\mathrm{ec}}}{2\tau _{\mathrm{ex}}}}\right) \end{aligned}$$

where $\textit{K}_{0}$ describes the variance of the field inhomogeneities due to the susceptibility changes, $\gamma$ is the gyromagnetic ratio, $\textit{t}_{\mathrm{ec}}$ is the time between echoes, and ${\tau }_{\mathrm{ex}}$ is the exchange time. As in Eq. 1, $\textit{T}_{20}$ is the intrinsic $\textit{T}_{2}$ of blood, which describes the relaxation when no exchange occurs and is the limiting case as the CPMG echo interval goes to zero.

An alternative model to describe the $\textit{T}_{2}$ shortening due to deoxygenation was introduced by Jensen and Chandra [31], who found that the relaxation rate was given by

3 $$\begin{aligned} \frac{1}{T_2} = \frac{1}{T_{20}}+ G_0 \frac{\gamma ^2 r_{\mathrm{c}}^2}{2D} F\left( \frac{2D t_{\mathrm{ec}}}{r_{\mathrm{c}}^2}\right) \end{aligned}$$

where $$F(x) = \frac{1}{\sqrt{\pi }} \int _0^\infty \frac{{\mathrm{e}}^{-y}}{\sqrt{y}} \left[ 1-\frac{1}{xy} \tanh {xy}\right] {\mathrm {d}}y.$$

This model describes protons diffusing through an inhomogeneous field, where $\textit{r}_{\mathrm{c}}$ is the size of the field inhomogeneities, *D* is the diffusion coefficient of water, assumed to be $2\times 10^{-9}~\text{m}^{2}/\text{s}$, and $\textit{G}_{0}$ is the mean squared magnitude of the field inhomogeneities.

The equations for the two models were fitted to the $\textit{T}_{2}$ values measured with 13 inter-echo intervals using least squares. This allowed us to estimate the parameters $\textit{T}_{20}$, ${\tau }_{\mathrm{ex}}$/$\textit{r}_{\mathrm{c}}$, and $\textit{K}_{0}$/$\textit{G}_{0}$. To quantify the agreement with the data, the sum of squared residuals (SSR) was calculated.

## Results

**Fig. 2:**
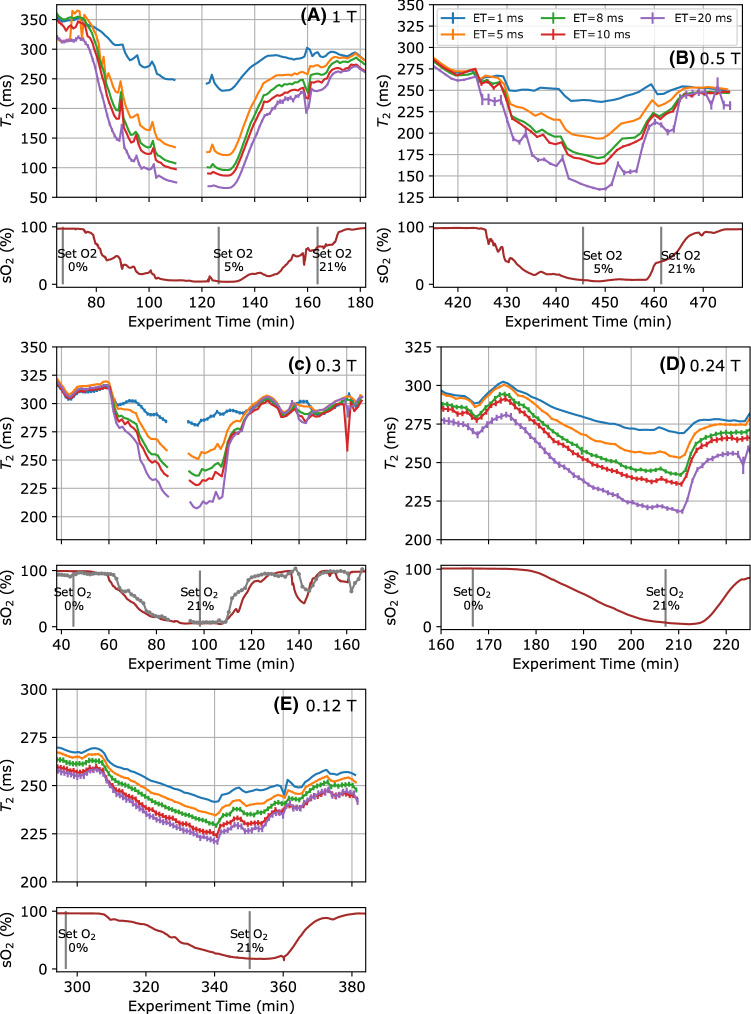
Measured $\textit{T}_{2}$ values for blood during oxygenation cycles at different field strengths. Below each $\textit{T}_{2}$ measurement, the optical measurements of oxygenation are included. Error bars show the standard deviation of the $\textit{T}_{2}$ reported by the curve fitting algorithm at B_0_ field strengths of (**A**) 1 T, (**B**) 0.5 T, (**C**) 0.3 T, (**D**) 0.24 T and (**E**) 0.12 T. The same colours for different CPMG echo intervals (ET) are used in all graphs. The grey trace in **C** shows an MR based estimate of oxygen saturation using $\textit{T}_{2}$ measurements based on multiple inter-echo intervals

The experiments at each field strength produced a series of correlated $\textit{T}_{2}$/${\textit{s}}\text{O}_{2}$ data points measured with five different inter-echo intervals. These are shown in Fig. 2, with markers indicating different steps in the experimental protocol. The oxygenation level from the optical sensor is plotted in the lower part of the graph, and the fitted $\textit{T}_{2}$ values in the upper part. Across all the field strengths in Fig. 2, a clear decrease is visible in the $\textit{T}_{2}$ measurements as the oxygen saturation falls. This recovers as the oxygen saturation increases back to 100%.

In addition, there is a clear separation between the $\textit{T}_{2}$ values measured with different inter-echo intervals which grows as the oxygen saturation falls. The change in $\textit{T}_{2}$ is larger with longer inter-echo intervals, and there is also a significant decrease in the size of the change as the $\textit{B}_{{0}}$ field decreases. The change in $\textit{T}_{2}$ between the oxygenated and deoxygenated blood at 1 T is up to 200 ms, while at 0.12 T, the range is 10–20 ms.

Some of the field strengths have an additional spread in the $\textit{T}_{2}$ measurements that depends on inter-echo interval. This is visible as spacing between the $\textit{T}_{2}$ traces which is still present when the blood is fully oxygenated and is constant over the length of each experiment, although this effect is less significant than the change due to oxygenation.

Furthermore, there are some transient changes in $\textit{T}_{2}$ that are not correlated with the oxygen saturation, for example at the start of Fig. 2D, E. These variations in $\textit{T}_{2}$ of up to 15 ms are correlated with fluctuations in the flow rate of up to 5–10% (as measured by PGSE), which occurred as the flow rate through the system balanced after the pump and bag heights were adjusted. At the lowest fields we have measured, flow fluctuations may be dominating the oxygenation/$\textit{T}_{2}$ contrast, which becomes small at these field strengths.

In addition, we also observed a decreasing trend in $\textit{T}_{2}$ over the course of the experiments. This decrease is roughly linear over the course of the experiment, and unlike the changes due to oxygen saturation, does not appear to be dependent on the inter-echo interval. The 0.5 T data were collected directly after the 1 T experiment, with the same blood sample, and has a lower initial $\textit{T}_{2}$, which we believe is due to the same effect. To identify the cause of the decrease, samples of blood from the circuit were taken and separated by centrifuge. UV/Vis spectra of the separated plasma showed an increasing concentration of haemoglobin over time.

**Table 1:** Best fit values for $\textit{R}_{{2}}$ = $R_{20} + K(\textit{t}_{\mathrm{ec}}, \textit{B}_{{0}}) {\textit{s}}\text{O}_{2}^2$

$\textit{B}_{{0}}$ field	*K* (1 ms)	*K* (5 ms)	*K* (8 ms)	*K* (10 ms)	*K* (20 ms)
0.24	0.28 ${\pm }$ 0.03	0.48 ${\pm }$ 0.04	0.59 ${\pm }$ 0.05	0.66 ${\pm }$ 0.06	0.85 ${\pm }$ 0.07
0.3	0.35 ${\pm }$ 0.02	0.83 ${\pm }$ 0.03	1.09 ${\pm }$ 0.04	1.22 ${\pm }$ 0.04	1.67 ${\pm }$ 0.05
0.5	0.51 ${\pm }$ 0.04	1.57 ${\pm }$ 0.07	2.23 ${\pm }$ 0.09	2.51 ${\pm }$ 0.09	3.66 ${\pm }$ 0.17
1.0	1.29 ${\pm }$ 0.03	5.08 ${\pm }$ 0.11	7.17 ${\pm }$ 0.14	8.12 ${\pm }$ 0.16	11.20 ${\pm }$ 0.23

At 0.12 T, the data did not agree with a quadratic curve, so the fit parameters are not reported

**Fig. 3:**
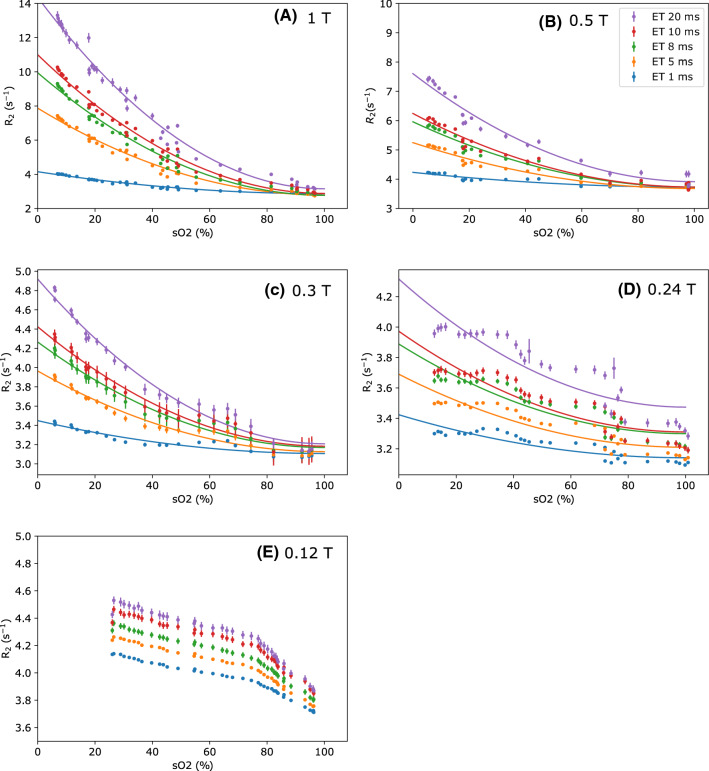
Points indicate $\textit{R}_{{2}}$ during oxygenation ramps plotted against measured ${\textit{s}}\text{O}_{2}$, at different field strengths. Curves show best fit to quadratic models, with best fit parameters in Table 1. At the lowest field, the quadratic curve is no longer a good fit to the data. Error bars show the standard deviation of the estimate of $\textit{T}_{2}$ reported by the fitting algorithm. The same colours are used in all graphs

${R}_{2}$ *fitting* Because of the rapid change in the oxygen saturation during reoxygenation, only points measured during the deoxygenation sections were extracted for further analysis. These data points are shown in Fig. 3, along with the results of fitting these points to a quadratic curve. The fit values are shown in Table 1. At 1 T, 0.5 T and 0.3 T the $\textit{R}_{{2}}$ values show good agreement with the quadratic curve. At 0.24 T, the trend is more difficult to determine due to variability in $\textit{R}_{{2}}$. At 0.12 T, the change in $\textit{R}_{{2}}$ is small and difficult to observe and does not agree with a quadratic fit. In the lower $\textit{B}_{{0}}$ fields, the data appear more variable, as the change due to oxygenation becomes smaller. Other processes that influence $\textit{T}_{2}$ have a larger effect on our $\textit{R}_{{2}}$ results, and can make it difficult to isolate the oxygenation effect. This shows that this contrast is less sensitive at low fields, with $\textit{T}_{2}$ decreases of the order of 5–10 ms, rather than 100 ms at 1 T.

**Fig. 4:**
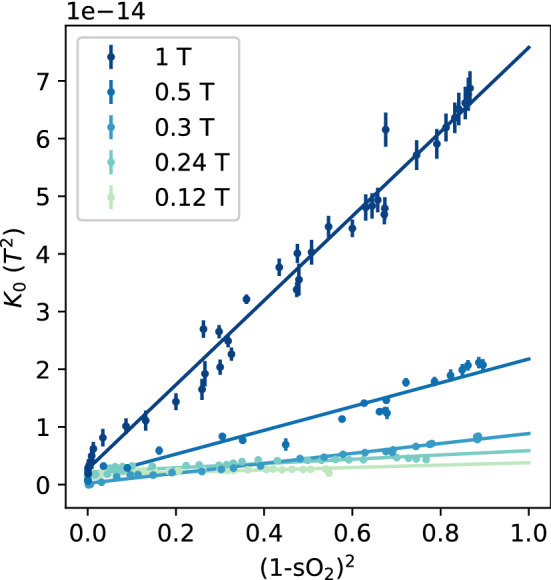
Dependence of $\textit{K}_{0}$ on ${\textit{s}}\text{O}_{2}$ at B_0_ field strengths of (**A**) 1 T, (**B**) 0.5 T, (**C**) 0.3 T, (**D**) 0.24 T and (**E**) 0.12 T. The $\textit{K}_{0}$ values were found by fitting to five $\textit{T}_{2}$ values measured with different CPMG echo intervals, as described in the text. Note that the 0.12 T data only extend to ${\textit{s}}\text{O}_{2}$ = 25%. Error bars show the standard deviation of the estimates of $\textit{K}_{0}$ from the fitting algorithm

The $\textit{R}_{{2}}$ values from Fig. 3 were also used to fit the exchange model equation to test the dependence of $\textit{K}_{0}$ on ${\textit{s}}\text{O}_{2}$. The Luz–Meiboom equation (Eq. 2) was fit to the $\textit{T}_{2}$ measurements at 5 inter-echo intervals to estimate a value of $\textit{K}_{0}$. These used a fixed value of ${\tau }_{\mathrm{ex}}={3.3}$ ms, as reported by Stefanovic [18], and ignored the effect of haematocrit. Plotting the $\textit{K}_{0}$ values against $(1-{\textit{s}}\text{O}_{2})^{2}$ yields a linear trend with $r^2$ correlation coefficients around 0.9, with the exception of the measurements at the lowest field (0.12 T). These are shown in Fig. 4, with the linear fit coefficients included in Table 2.

**Table 2:** The linear fit coefficients for the $\textit{K}_{0}$ parameter as a function of (1-${\textit{s}}\text{O}_{2}$)^2^ at the different $\textit{B}_{{0}}$ field strengths used in the experiments

$\textit{B}_{{0}}$ field (T)	Slope ($10^{-14}~\text{T}^2$)	Intercept ($10^{-14}~\text{T}^2$)	${r}^{2}$
0.12	0.21 ${\pm }$ 0.03	0.17 ${\pm }$ 0.01	0.57
0.24	0.37 ${\pm }$ 0.03	0.22 ${\pm }$ 0.01	0.81
0.3	0.86 ${\pm }$ 0.02	0.02 ${\pm }$ 0.01	0.99
0.5	2.06 ${\pm }$ 0.10	0.12 ${\pm }$ 0.05	0.96
1.0	7.31 ${\pm }$ 0.16	0.27 ${\pm }$ 0.08	0.98

**Fig. 5:**
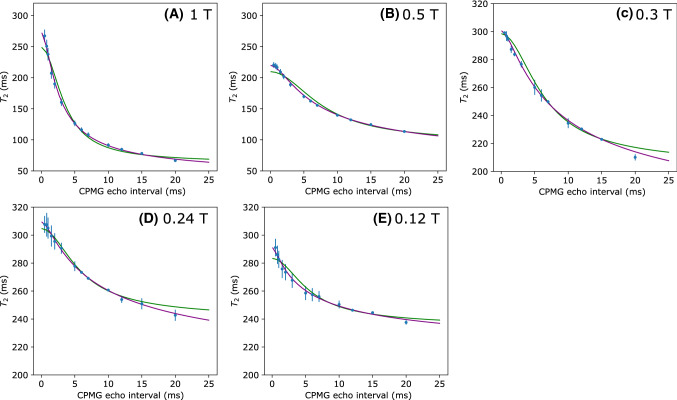
$\textit{T}_{2}$ measurements for deoxygenated blood samples as a function of CPMG inter-echo interval at B_0_ field strengths of (**A**) 1 T, (**B**) 0.5 T, (**C**) 0.3 T, (**D**) 0.24 T and (**E**) 0.12 T. Best fit curves from the exchange model (Green) and the diffusion model (Purple) are also shown. Note the different scales for 1 T and 0.5 T. Error bars show the standard deviation of the estimate of $\textit{T}_{2}$

*Model fitting* After the blood reached a deoxygenated state, CPMG experiments with a finer range of inter-echo intervals were measured. These results are shown in Fig. 5 which shows the $\textit{T}_{2}$ values decrease as the inter-echo interval increases. The $\textit{T}_{2}$ drops sharply in the range between 0.5 and 5 ms but then decreases more slowly as the inter-echo interval increases. As expected, measurements at 0.5 T and 1 T show a much larger decrease when compared to the measurements at 0.3 T and below. The 0.5 T results in this figure were measured using blood from the 1 T experiment, and have a lower initial $\textit{T}_{2}$ due to the breakdown of blood in the circuit.

**Table 3:** Best fit parameters for Luz–Meiboom and Jensen and Chandra models at the different field strengths

$\textit{B}_{{0}}$ field (T)	$\textit{K}_{0}$ ($10^{-14}~\text{T}^2$)	${\tau }_{\mathrm{ex}}$ (ms)	$\textit{T}_{20}$ (ms)	SSR ($\text{ms}^2$)	$\textit{G}_{0}$ ($10^{-14}~\text{T}^2$)	$\textit{r}_{\mathrm{c}}$ ($\upmu \text{m}$)	$\textit{T}_{20}$ (ms)	SSR (${\text{ms}^2}$)
0.12	0.59 ${\pm }$ 0.1	1.8 ${\pm }$ 0.3	283 ${\pm }$ 3	177	1.3 ${\pm }$ 0.5	2.3 ${\pm }$ 0.4	291 ${\pm }$ 6	34
0.24	0.65 ${\pm }$ 0.06	1.9 ${\pm }$ 0.18	305 ${\pm }$ 3	89	1.0 ${\pm }$ 0.16	3.3 ${\pm }$ 0.4	309 ${\pm }$ 4	13
0.3	0.99 ${\pm }$ 0.02	2.30 ${\pm }$ 0.08	298 ${\pm }$ 0.5	183	1.39 ${\pm }$ 0.04	3.7 ${\pm }$ 0.12	300 ${\pm }$ 0.6	35
0.5	2.45 ${\pm }$ 0.05	3.55 ${\pm }$ 0.08	210 ${\pm }$ 2	498	3.5 ${\pm }$ 0.1	4.6 ${\pm }$ 0.12	220 ${\pm }$ 3	34
1.0	8.4 ${\pm }$ 0.3	2.1 ${\pm }$ 0.1	248 ${\pm }$ 6	1199	11.6 ${\pm }$ 0.6	3.6 ${\pm }$ 0.19	272 ${\pm }$ 9	120

Equations 2 and 3 for the exchange model and the diffusion model, respectively, were then fit to these $\textit{T}_{2}$ values (Fig. 5), and the best fit parameters and residuals are included in Table 3. Both theoretical curves show good agreement with the data points. Generally, the fits to the diffusion model also show a lower SSR than the exchange model. With the exception of the 0.5 T result, the fit parameters give results of ${\tau }_{\mathrm{ex}}$ = ${2.17\pm 0.06}$ ms and $\textit{r}_{\mathrm{c}}$ = ${3.58\pm 0.08}~\upmu \text{m}$ (weighted mean across field strengths) that are concordant with the literature.

## Discussion

${T}_{2}$ and $s\text{O}_{2}$ Our results show that changes in blood $\textit{T}_{2}$ due to oxygen saturation are still visible at low fields. At 0.3 T and 0.24 T, the relaxation change becomes much smaller, but is still detectable; of the order of 10 ms. At the fields below 0.3 T, the relaxation change was small and potentially overwhelmed by other confounding factors (see below). This is important for informing the design of future low-field MR systems if they are aiming to use this contrast for physiological monitoring.

In line with literature measurements at higher fields ($>1.5$ T), we observed that the CPMG echo interval also affects the blood $\textit{T}_{2}$ as it is deoxygenated, which suggests that the mechanism for decreasing $\textit{T}_{2}$ remains the same. The splitting between the $\textit{T}_{2}$ measurements with different echo intervals in Fig. 2 decreases at the lowest fields we used, with the 0.12 T and 0.24 T results showing a smaller change in $\textit{T}_{2}$ due to inter-echo interval as a function of oxygen saturation. Figure 3 shows that the increase in $\textit{R}_{{2}}$ as ${\textit{s}}\text{O}_{2}$ falls appears to be proportional to $(1-{\textit{s}}\text{O}_{2})^{2}$, but at 0.24 T, where induced field inhomogeneity becomes smaller, the change in $\textit{R}_{{2}}$ appears more linear. It is also possible that a linear change in $\textit{R}_{{2}}$ occurs due to deoxy-haemoglobin acting like a paramagnetic contrast agent, which may be more significant than the effect of diffusion through $\textit{B}_{{0}}$ inhomogeneity at short inter-echo intervals.

The absolute $\textit{T}_{2}$ values measured in these experiments are generally higher than those already reported in the literature at higher fields $({>1.5}$ T), with $\textit{T}_{2}$ values around 300 ms compared to the 200 ms observed by Stefanovic and Pike or Lu et al. [14, 18]. Brooks et al. and Wright et al. found blood $\textit{T}_{2}$ was around 250 ms at 1.0 T and 1.5 T which is closer to our measurements [12, 24]. There are a number of factors which could explain this observation, including the lower haematocrit in the samples used in our experiments (0.33–0.38 rather than 0.4), which is known to affect blood relaxation [12, 21]. There is also variation in the blood temperature used in the literature. Some studies such as Stefanovic and Pike, or Meyer et al. used 22 ${}^{\circ }\text{C}$, the ambient temperature in the magnet [16, 18]. Brooks et al. and Wright et al. measured the blood at 37 ${}^{\circ }\text{C}$, which is its temperature in vivo, and may explain why their $\textit{T}_{2}$ values are closer to ours [12, 24]. Our experiments measured the blood at 27–30${}^{\circ }\text{C}$, which is as close as we could get to body temperature without increasing the temperature of the blood beyond body temperature at the oxygenator. In addition, some previous studies have used different preparation methods for the blood samples such as washing and resuspending the red blood cells, that may affect their results. In contrast, our experiments used leucocyte reduced whole blood which may be more representative of in vivo blood than the washed and resuspended red blood cells, as it maintains the remaining plasma fraction component. Other studies have used conventional MRI systems and imaging pulse sequences, which may affect the $\textit{T}_{2}$ values they obtain when compared to the non-imaging CPMG sequence we used.

The relative change in $\textit{T}_{2}$ between oxygenated and deoxygenated blood are of a similar scale to the changes reported in the low field literature. Gomori found a decrease of approximately 20 ms at the field strengths used here, although they indicate a large degree of uncertainty in their results [26]. In contrast, Brooks et al. suggest that there is still a large difference in the relaxation rate when comparing samples of deoxygenated and oxygenated blood which continues down to the lowest field they measure. They observe a decrease from 250 to 125 ms even at a field of 0.02 T [24]. This is not supported by our results which showed that the $\textit{T}_{2}$ change between oxygenated to deoxygenated states decreased with field strength. This may be due to the different blood cell preparation methods used by Brooks et al., or differences in experimental technique.

The relationship between $\textit{K}_{0}$ and $(1-{\textit{s}}\text{O}_{2})^2$ has also been observed in the literature at 1.5 T [18] and 3 T [19]. Our results in Fig. 4 and Table 2 also show this relationship down to 0.3 T, but the experiments at the lowest fields have a very small slope term. With the exception of the lowest field strength (0.12 T), the slope terms are proportional to $\textit{B}_{{0}}^2$. Extrapolating the slope terms from Table 2 to 1.5 T gives a value of $\textit{K}_{0}= (1.8\pm 0.6)\times 10^{-13}~\text{T}^2$ $(1-{\textit{s}}\text{O}_{2})^2$ at 1.5 T, which is lower than the value given by Stefanovic and Pike of $(3.1\pm 0.2)\times 10^{-13}~\text{T}^2$ [18]. This difference may be related to the different ranges of oxygenation we used, or the $\textit{B}_{{0}}$ field strengths.

*Confounding factors* Some of the experiments showed that $\textit{T}_{2}$ appears to have a dependence on CPMG echo interval that still exists when the blood is fully oxygenated, which does not agree with theory. This additional decrease in $\textit{T}_{2}$ as inter-echo interval increases is relatively static over the course of the experiment, which suggests that it is a systematic effect in these measurements. In particular, it is the same before and after the deoxygenation cycle, so this spread should not alter the $\textit{T}_{2}$ changes caused by oxygen saturation. One process that could explain this spread is diffusion of protons through background $\textit{B}_{{0}}$ field gradients produced by the magnet. This inhomogeneity is generated by the design of the magnet which is designed to operate at 1.5 T, higher than the fields we worked at. The $\textit{B}_{{0}}$ inhomogeneity also changes as $\textit{B}_{{0}}$ was charged to different field strengths. The homogeneity could be improved by magnet shimming for the different $\textit{B}_{{0}}$ field strengths. Another process that could explain this is a mismatch between the susceptibility of the red blood cells and plasma, which can produce $\textit{B}_{{0}}$ field gradients while the blood is oxygenated.

The increase in plasma haemoglobin concentration over the course of the experiments suggest that a small amount of haemolysis occurs as the blood flows through the circuit. This may be a contributing factor to the linear decrease in $\textit{T}_{2}$ over time that we observed in some experiments. While Gomori found that complete hemolysis caused an increase in $\textit{T}_{2}$ [26], the decrease in $\textit{T}_{2}$ over the course of these experiments is still visible when the blood is fully oxygenated, and could be due to a low but increasing concentration of haemoglobin that lowers the $\textit{T}_{2}$ of water in the plasma. Another factor may be the formation of met-haemoglobin over time due to the oxidation of haemoglobin, which may affect $\textit{T}_{2}$. While these effects makes it more difficult to rigorously investigate how inter-echo interval affects the $\textit{T}_{2}$ change due to oxygenation, the decrease and recovery in $\textit{T}_{2}$ are still visible in the results at low field.

There are also additional factors that could create uncertainty in the blood itself. Differences in the haematocrit between samples for each field strength were not taken into account in these experiments. iStat measurements of the haematocrit of the blood samples showed that they were all between 0.33 and 0.38, following dilution with the anticoagulant solution during blood collection. While lower than physiological values, the small range means that it should not have a significant effect on the results found comparing the different fields.

Temperature is also known to affect $\textit{T}_{2}$ values. In these experiments, temperature was continuously monitored and controlled by flowing water from a 37 ${}^{\circ }\text{C}$ water bath through the oxygenator. Despite this, there were fluctuations in the temperature of up to 4 ${}^{\circ }\text{C}$ over the course of the experiments, mainly due to changes in flow through the oxygenator. As noted above, these fluctuations caused variations of up to 15 ms in $\textit{T}_{2}$, however, they did not appear to affect the difference between $\textit{T}_{2}$ measured with different CPMG echo intervals.

*Models and mechanisms* The experiments with varying inter-echo intervals show that the Jensen and Chandra diffusion model describes the change in $\textit{T}_{2}$ due to the inhomogeneous field in blood more accurately than the exchange model, producing lower residuals in Table 3. This was true for all the $\textit{B}_{{0}}$ field strengths tested in these experiments, suggesting that the diffusion model provides a better description of the mechanism behind this effect than the chemical exchange model. Some previous studies in the literature have found this conclusion at field strengths of 1.5 T [18], 3 T [19] and 7 T [20]. However, the improvement has been found to be marginal in other studies [32] and some studies have found the opposite conclusion [22].

When comparing the best fit curves in Fig. 5, there is relatively little difference in the predictions of the two models. Generally, the difference between the curves is within the range of uncertainty of our system, so the exchange model cannot be ruled out by our experiments. Li and Zijl showed that at higher fields the change in blood $\textit{T}_{2}$ can be explained by the combination of exchange and diffusion, with the relative contribution to relaxation dependent on the CPMG echo interval [23]. Regardless of the mechanism, our results show that the simpler exchange model is an still an appropriate approximation for describing the behaviour of the $\textit{T}_{2}$ contrast in blood at low field.

The estimated parameters ${\tau }_{\mathrm{ex}}$ = $2.1\pm 0.1$ ms and $\textit{r}_{\mathrm{c}}$ = $3.5\pm 0.1$ $\upmu \text{m}$ from these experiments are within the range reported in the literature (${\tau }_{\mathrm{ex}}$ = 0.6 to 10 ms, $\textit{r}_{\mathrm{c}}$ = 2.7 to 4.3 $\upmu \text{m}$) [18, 24, 26, 28]. There do not appear to be any trends related to the field strength, which should only affect the $\textit{K}_{0}$ and $\textit{G}_{0}$ terms (as the ${\textit{s}}\text{O}_{2}$values are all low). As there are only a small number of samples, meaningful conclusions cannot be drawn about the meaning of these parameters from this data.

*Oximetry with NMR* In previous examples in the literature, a calibration curve has been used to obtain ${\textit{s}}\text{O}_{2}$ from $\textit{T}_{2}$ measurements [12, 14]. In our in vitro experiments, however, this method does not work well due to the drifts in $\textit{T}_{2}$ from other changes in the blood, such as the release of haemoglobin or temperature change, meaning the calibration curve is no longer accurate. For a more robust estimate of oxygen saturation, a method to estimate the field inhomogeneity ($\textit{K}_{0}$ in the Luz–Meiboom equation) from $\textit{T}_{2}$ measured with different inter-echo intervals was explored [28]. As the measured $\textit{T}_{2}$ is dependent on CPMG echo interval, making $\textit{T}_{2}$ measurements with multiple inter-echo intervals extracts more information, allowing the effects from drifts in $\textit{T}_{20}$ and $\textit{K}_{0}$ to be separated. A calibration curve to relate $\textit{K}_{0}$ and ${\textit{s}}\text{O}_{2}$ should then provide a more robust estimate of oxygen saturation. This method is demonstrated on the 0.3 T data in the grey trace in Fig. 2C. It agrees with the optical data, although rapid dips in ${\textit{s}}\text{O}_{2}$ appear slightly differently, which may reflect changes in ${\textit{s}}\text{O}_{2}$ in the time between the optical and the 5 CPMG measurements.

Combining $\textit{T}_{2}$ measurements with different CPMG echo intervals is effectively the same as a method presented by Varghese et al. [28], who found this method gave improved accuracy for blood oximetry in the heart. This method may be useful for non-imaging relaxometry systems, which can be more flexible in the range of inter-echo intervals that can be collected, although it is also sensitive to background $\textit{B}_{{0}}$ field inhomogeneity, as it relies on the dependence of blood $\textit{T}_{2}$ on inter-echo interval.

## Conclusions

These experiments show that the $\textit{T}_{2}$ contrast due to deoxy-haemoglobin in red blood cells is still visible at $\textit{B}_{{0}}$ fields below 1 T. While the size of the change decreases proportional to $\textit{B}_{{0}}^2$, the $\textit{T}_{2}$ drop for deoxygenated blood can be up to 20ms for extremely low oxygen saturations at a field of 0.12 T. Measurements on the dependence of $\textit{T}_{2}$ on CPMG echo interval agree with existing results in the literature that the diffusion model explains the contrast more accurately than the exchange model, but that the difference between the two models is not very significant. Changes in blood $\textit{T}_{2}$ due to deoxygenation should be measurable with MR systems which operate at lower field strengths such as 0.3 T or 0.5 T, as well as portable low field relaxometry devices at these field strengths, although precise quantification of oxygenation at lower $\textit{B}_{{0}}$ field strength below 0.3 T may be challenging.
